# Fast Waves at the Base of the Cochlea

**DOI:** 10.1371/journal.pone.0129556

**Published:** 2015-06-10

**Authors:** Alberto Recio-Spinoso, William S. Rhode

**Affiliations:** 1 Instituto de Investigación en Discapacidades Neurológicas, Universidad de Castilla-La Mancha, Albacete, Spain; 2 Department of Neuroscience, University of Wisconsin, Madison, Wisconsin, United States of America; University of South Florida, UNITED STATES

## Abstract

Georg von Békésy observed that the onset times of responses to brief-duration stimuli vary as a function of distance from the stapes, with basal regions starting to move earlier than apical ones. He noticed that the speed of signal propagation along the cochlea is slow when compared with the speed of sound in water. Fast traveling waves have been recorded in the cochlea, but their existence is interpreted as the result of an experiment artifact. Accounts of the timing of vibration onsets at the base of the cochlea generally agree with Békésy’s results. Some authors, however, have argued that the measured delays are too short for consistency with Békésy’s theory. To investigate the speed of the traveling wave at the base of the cochlea, we analyzed basilar membrane (BM) responses to clicks recorded at several locations in the base of the chinchilla cochlea. The initial component of the BM response matches remarkably well the initial component of the stapes response, after a 4-μs delay of the latter. A similar conclusion is reached by analyzing onset times of time-domain gain functions, which correspond to BM click responses normalized by middle-ear input. Our results suggest that BM responses to clicks arise from a combination of fast and slow traveling waves.

## Introduction

The arrival of sounds at the mammalian ear sets off a chain of signal transformations. Pressure waves traveling in the air are converted into vibrations of the middle ear bones. Such vibrations, specifically those of the stapes, serve as the mechanical input to the hearing organ, the cochlea. Stapes vibrations induce movements of the cochlear fluids and initiate a displacement wave on the basilar membrane (BM) that travels from the base, near the stapes, to the distal end of the cochlea, or apex. We largely owe this description of cochlear mechanics to Georg von Békésy [1].

Von Békésy’s experiments with brief-duration stimuli indeed indicated that basal BM regions move earlier than more apical ones, contradicting Helmholtz’s theory [2] that all cochlear regions start moving at the same time and without any delay. The main characteristics of the progressive delays found by von Békésy have been confirmed in the cochleae of live animals, albeit via indirect estimates of BM motion (e.g., [3, 4]). Additional confirmation has come from direct measurements of BM vibrations (e.g., [5]), performed mostly at the base of the cochlea. The consensus is that, following middle-ear displacements, locations along the BM start to move after a given delay. This delay, the difference between the times of vibrations onsets of the stapes and the BM, is independent of stimulus frequency and increases as a function of distance to the stapes. We will refer to this time difference as the *signal-front delay* [6, 7].

Signal-front delays derived from responses to clicks of auditory nerve fibers (ANFs) vary little for characteristic frequencies (CF: the most sensitive frequency) in the basal half of the cochlea [3]. In general, delays of mechanical or neural responses appear to increase with distance to the stapes and—at least at the base of cochlea [5]—are very short, in the order of a few tens of microseconds. Some authors have judged such signal-front delays as incompatible with a traditional (slow) traveling wave (e.g., [8–10]).

Because of certain issues with the above estimates of signal-front delays, we analyzed mostly unpublished measurements of chinchilla BM responses to clicks. These responses originated either from at least two sites in the same cochlea or from sites at the apical end of the first cochlear turn in different animals (CFs in the 5.5–7 kHz range). Two types of analyses were performed: One consists of plotting together BM and stapes responses to clicks in the same cochlea and comparing their onsets; the other consists of obtaining a gain function, which equals the BM response to clicks normalized by middle-ear input. Results from the analyses indicate that signal-front delays are only ≈ 4 μs, equivalent to one sample in our data acquisition system, and do not vary across different sites of the first cochlear turn. (The 4-μs signal-front delay is a fraction of previous delay estimates in the chinchilla [5, 11].) We interpret our results as indicating the existence of two traveling waves: a fast one, which travels at the speed of sound in water, and a slow one, similar to the one described by von Békésy.

## Methods

Experiments were performed on 11 chinchillas (average weight: 500 g). In nine of the 11 chinchillas, recordings were made in at least two locations. Animals were used at the University of Wisconsin–Madison, USA. The care and use of animals in this study were approved by the Animal Care and Use Committee of the University of Wisconsin (protocol number: A-53-5400-M00457). Details of surgical and recording methods for this type of experiment are given elsewhere (e.g., [5, 12, 13])

### Animal preparation

Animals were anesthetized using an initial dose of sodium pentobarbital (75 mg kg−1, i.p., Sigma-Aldrich) and additional smaller doses were given as needed to maintain the animal in a deeply areflexive state. All animals were tracheotomized and intubated, but forced ventilation was usually unnecessary. Normal body temperature was maintained at 37°C via a heating pad servo-controlled by a rectal probe. The left pinna was resected and the bulla was widely opened. A silver-wire electrode was placed on or near the round window to record compound action potentials (CAPs) evoked by tone bursts at frequencies usually between 500 Hz and 16 kHz. CAP audiograms were estimated manually using oscilloscope recordings. Experiments were finished if there was an increase of more than 10–15 dB in thresholds. All data presented here originate from non-linear preparations, as concluded from the compressive growth rates of responses to CF tones and click stimuli. A small hole made in the basal turn of the otic capsule allowed direct visualization of the basilar membrane and placement of a few micro-beads (average diameter: 25 μm) to serve as reflecting targets for the displacement-sensitive heterodyne laser interferometer [12]. BM vibrations were measured after covering the hole in the otic capsule with a small window made from cover slip glass. Vibrations were also recorded from micro-beads placed on the stapes, near the incudo-stapedial joint, or on the umbo of the tympanic membrane. At the end of the experiments each animal was euthanized with a high dose of sodium pentobarbital.

### Acoustic stimulation

Acoustic stimuli were generated using a personal computer in conjunction with a 16-bit digital-to-analog converter and an attenuator system (Tucker-Davis Technologies, Alachua, Fla., USA) at sampling rates of 200 kHz. Stimuli were presented closed-field from a reverse-driven condenser microphone cartridge (Brüel & Kjær 4134 with square-root compensation, Nærum, Denmark). Sound-pressure levels (measured in dB SPL) were monitored within 2 mm of the tympanic membrane using a probe tube microphone.

Stimuli used in this project include clicks and single tones, which were used only to calibrate click levels. Durations of tones and click stimuli were 30 ms and 10 μs, respectively. Tone levels are expressed in dB SPL. Click levels are expressed as peak-equivalent SPL (dB pSPL) and were determined from middle-ear velocity responses to clicks and tones: the pSPL of a click corresponds to the SPL of a 1 kHz-tone with the same amplitude vibration.

### Data processing

Signals from the laser interferometer were sampled at a 250 kHz sampling rate using a 16-bit data acquisition card (Analogic Fast-16, USA). By fitting a sinusoidal function of a given frequency to a response waveform, amplitude and phase responses were obtained from BM and middle ear vibrations evoked by single tones. BM and middle ear responses to clicks were analyzed using Fast Fourier transform (FFT) routines available in MATLAB (Natick, Mass., USA). Akin to the transfer function of linear systems, gain functions were routinely estimated. These consists of the ratio, in the Fourier domain, of BM to middle-ear responses. CFs reported here were obtained from gain functions evaluated at the lowest available intensity level. Distances between the recording sites and the stapes were estimated using cochlear map equations [14] and assuming a BM length of 20.1 mm.

*Time-domain gain functions*, *h*(*t*), were defined in this paper as the click response of the BM normalized by that of the middle ear and were computed using a standard deconvolution technique. FFTs of BM, *BM*(*ω*), and middle ear, *ME*(*ω*), responses to clicks were obtained and the former FFT divided by the latter. The instantaneous gain function equals the inverse FFT, F^−1^{ }, of the aforementioned calculation:

$$h(t)={\text{F}}^{-1}\;\left\{\frac{BM(\omega )}{ME(\omega )}\right\}$$ (1)

where *ω* = 2*πf*, $j=\sqrt{-1}$, and *f* represents frequency (in Hz). A test of causality was also performed on *h*(*t*), The real and imaginary parts of a causal system are related by a Hilbert transform [15–17]. Let *H*(*ω*) be the Fourier transform of *h*(*t*), and equal to *X*(*ω*) + *jY*(*ω*). If, and only if, *h*(*t*), is a causal function then the following relation must be satisfied:

X(ω)=H{ω} (2)

where the operator *H*{ } denotes a Hilbert transform. Eq 2 was implemented using MATLAB’s *hilbert* function. The test of causality is important in verifying that the values of *h*(*t*) do not anticipate umbo or stapes input.

Instantaneous frequency representations were also estimated from time-domain gain functions using the analytic signal representation, as previously done by the authors for BM responses to clicks [5, 11]. Briefly, the analytic signal is a complex quantity whose real part equals the original waveform and whose imaginary part equals the Hilbert transform of the real part. The instantaneous frequency is defined as the derivative of the phase of the analytic signal.

## Results

**Fig 1:**
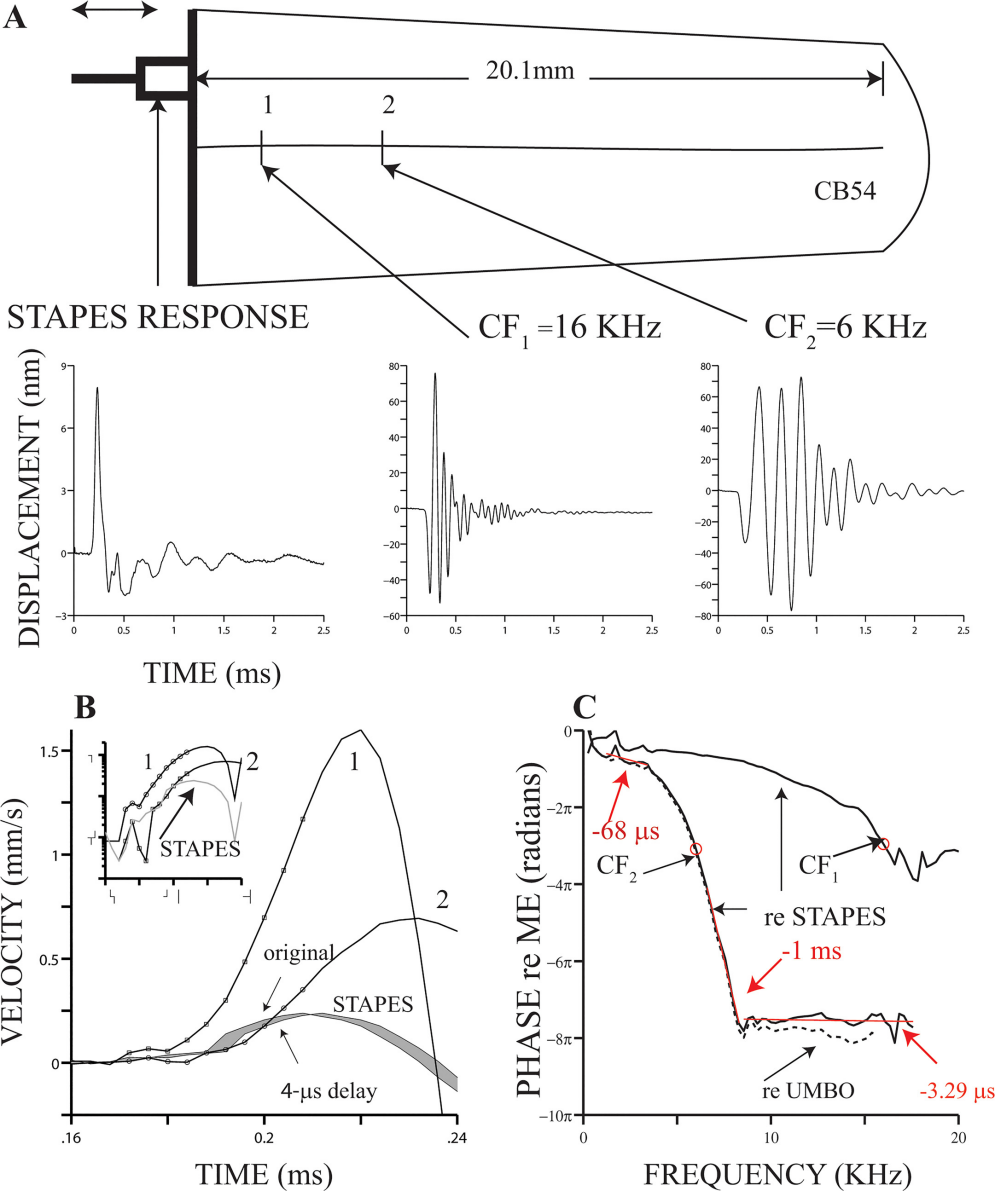
Middle-ear (ME) and basilar membrane (BM) vibrations evoked by acoustic clicks. The drawing at the top of panel (A) represents an uncoiled cochlea, whose length equals the mean value reported by [14]. The three plots at the bottom of panel (A), from left to right, represent stapes, BM at location 1 (CF = 16 kHz) and BM at location 2 (CF = 6 kHz) responses to 95-dB pSPL condensation clicks. Locations 1 and 2 are at a scale, following equations by [14]. Panel (B) exhibits velocity profiles of stapes and BM (locations 1 and 2) responses to 95-dB pSPL clicks. Waveforms displaying BM responses are shown inverted. Both original and shifted (4-μs) versions of the stapes responses are shown in (B). Inset in panel (B) displays in semi-logarithmic coordinates absolute values of the responses in main panel. Panel (C) displays phase-vs.-frequency functions from the click responses at locations 1 and 2. Phase functions were normalized relative to stapes (continuous lines) or umbo (dashed lines) motion. Continuous red lines in panel (C) represent linear fits to the phase-vs.-frequency segments indicated by the red lines. Negative numbers next to each red line represent the slope of each linear fit.

Mechanical responses of the middle ear (umbo and stapes) and the BM to acoustic clicks (at 95 dB pSPL) are displayed in Fig 1. BM responses were measured at two cochlear locations, 1 and 2. CFs estimated at locations 1 and 2 were 16 and 6 kHz, respectively. Unlike middle ear responses (lower left panel in Fig 1A), waveforms of BM responses to clicks have a bipolar shape (center and lower right panels in Fig 1A) that is characteristic of band-pass systems.

Onsets of stapes and BM responses in Fig 1A are shown in Fig 1B. To facilitate comparison between the onset times of stapes and cochlear motion, approximations to the first derivatives of the raw response waveforms are presented in Fig 1B. (The derivatives were approximated by finite differences, i.e., the difference between two adjacent samples, divided by 4 μs.) The negatives of the first derivative of BM responses are displayed in that figure; no polarity changes were made for the stapes response. In addition to the original version of the stapes response, a 4-μs delayed version of such response is also included in Fig 1B. Inspection of the stapes and the delayed version of the BM responses at location 2 in Fig 1B shows a remarkable similarity between the *initial segments* (small circles in Fig 1B) of these mechanical responses. The 4-μs delay corresponds to the signal-front delay as defined in the Introduction section.

BM click responses measured at location 1 exhibit a first oscillation whose amplitude is larger than the first oscillation measured at location 2 or at the stapes (Fig 1B), almost giving a false appearance of a delay between locations 1 and 2. The inset in Fig 1B, which is a semi-logarithmic plot of the absolute values of the curves in the main Fig 1B, shows a striking similarity between the delayed stapes vibrations and the motions recorded at locations 1 and 2, in spite of the differences in amplitudes.

Using the Fourier transform, we computed phase-vs.-frequency curves from the time-domain responses of Fig 1A. Phase functions in Fig 1C were expressed relative to stapes (continuous lines) and umbo (dashed lines) motion, as indicated by the arrows. Phase values decrease monotonically with frequency until they reach a plateau, one of which is indicated by a continuous red line in Fig 1C with a slope of -3.29 μs. The negative of the slope, the group delay, approximates the delay of certain features of a signal being filtered by a linear system [18]. The slopes of two other segments are also indicated in the same figure: -68 μs and -1 ms for the low- and high-frequency segments, respectively. Among the three aforementioned segments, only the plateau region exhibits a group delay that is similar in value to the 4-μs delay between the onsets of BM and stapes motions (Fig 1B). Our definition of signal-front delay is similar to Papoulis’ description of it (“…the delay of the beginning, or *front*, of a signal” [18]). For a linear system, this delay equals the high-frequency asymptotic slope of the phase function (see Eq 7–58 in [18]). It is difficult to ascertain if the delay around the plateau region in Fig 1C corresponds to the asymptotic values of the slope of the phase function, as the complete phase-vs.-frequency function is unknown. (For that reason, we did not evaluate the equation referenced above.) Nevertheless, both estimates are very similar.

One property of traveling waves, first shown by von Békésy [1] and confirmed by other authors (e.g., [5, 19]), can be observed in the phase curves in Fig 1B: there is a phase accumulation with frequency (as well as with distance along the BM). Another traveling wave property is the signal-front delay ([8]; see also comment by Peter Dallos in [20]). The 4-μs signal-front delay implied by the waveforms in Fig 1B approaches the delay of a wave traveling at the speed of sound in water, i.e., a compression wave.

**Fig 2:**
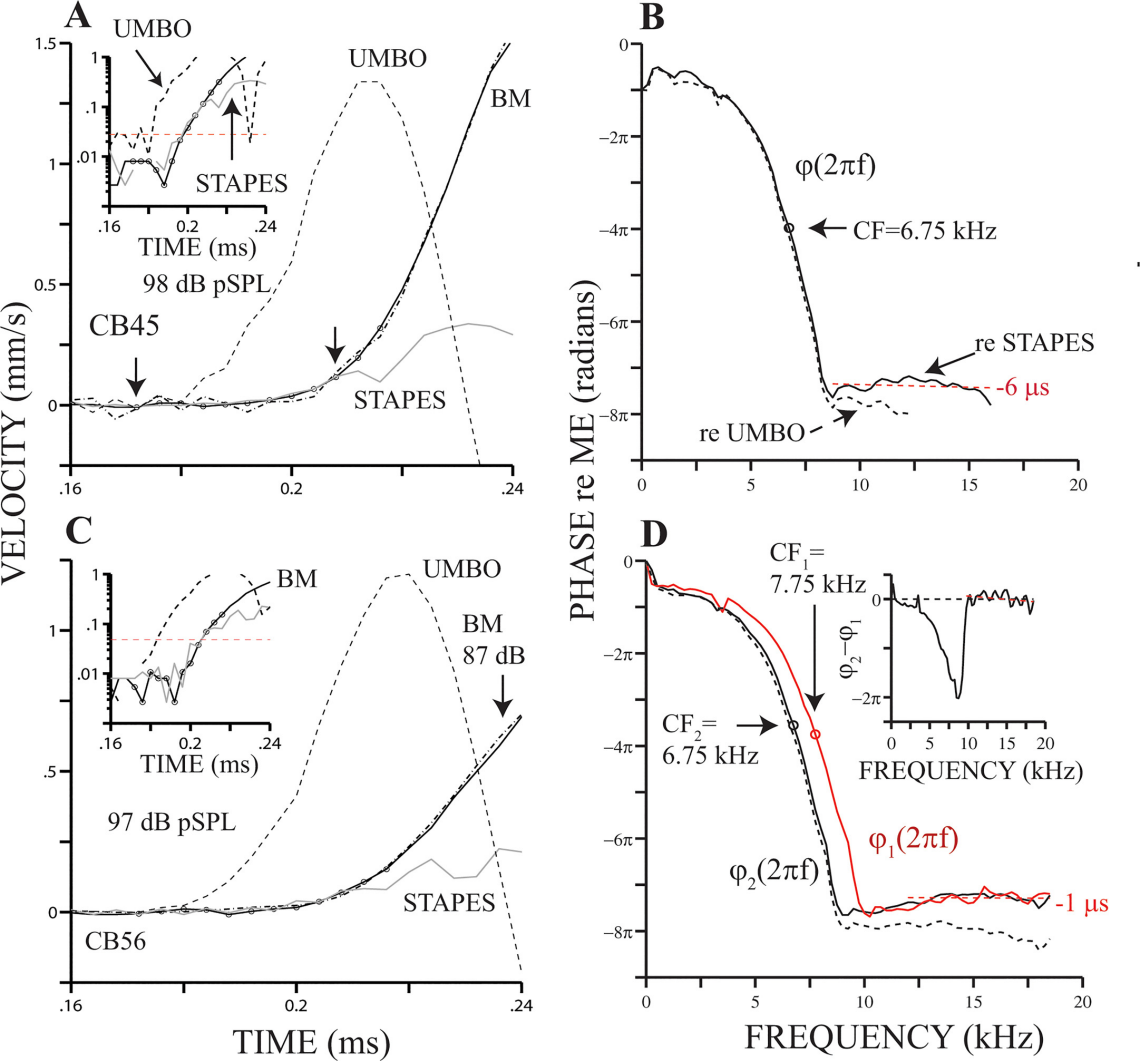
Onsets of stapes and BM responses to clicks are similar. Panels (A) and (C) display umbo, stapes and BM responses to clicks in the animal preparation indicated in each panel. Data are expressed in velocity units and BM responses are shown inverted. Stapes responses (gray lines) have been delayed by 4 μs. Continuous black lines with circles represent BM click response at the maximum possible level, as indicated in each panel. Dotted dashed lines display BM responses to clicks at the maximum level minus 10 dB. Insets in (A) and (B) display absolute values of stapes and BM responses in semi-logarithmic coordinates. Circles in the insets represent the so-called initial segment (see text). Black lines in (B) and (D) display phase-vs.-frequency curves, *φ*(2*πf*), of the responses in (A) and (C), respectively. Red trace in (D) represents a *φ*(2*πf*) measured from responses at another BM location in the same animal. Inset in (D) displays a local phase-vs.-frequency function equal to *φ*_2_(2*πf*) − *φ*_1_(2*πf*) [21].

Fig 2 displays middle ear and BM responses to clicks recorded in two cochleae. As in Fig 1, stapes responses (gray lines in Fig 2A and 2C) were also delayed by 4 μs. Stimulus levels appear in each of the panels in Fig 2 and correspond to the maximum values attainable with our system for a particular experiment (that is, no attenuation). By computing the correlation between the BM and stapes responses (continuous lines with circles and gray lines, respectively), we obtained a measure of the resemblance between the initial segments—i.e., the section delimited by the two small arrows in Fig 2A. Correlation coefficients, *r*, estimated from the initial segments of the BM and stapes responses in Fig 2A and 2C, equal 0.98 and 0.9, respectively (*p*-values ≤ 0.001, *t*-test). A high correlation value (*r* = 0.98, *p*≤ 0.001, *t*-test) was also estimated for the results in Fig 1B. In addition, dotted dashed lines in Fig 2A and 2C depict BM responses to clicks with levels 10 dB below the maximum values and appropriately scaled to compensate for the difference in stimulus levels (e.g., see Fig 2C). Results in Figs 1 and 2 indicate that the initial segments of stapes responses, delayed by 4 μs, and BM responses match remarkably well, even for 81–88 dB pSPL clicks.

Although results in Figs 1 and 2 do not indicate the actual times at which the stapes and BM start to vibrate, the results in those figures do suggest that the pure delay (i.e., the signal-front delay) between stapes and BM vibrations is 4 μs. The delay can be better appreciated in the semi-logarithmic plots shown as insets in Fig 2A and 2C. These insets display the absolute values of the BM and delayed stapes responses depicted in the corresponding main plots. Red dashed lines in the insets in Fig 2A and 2C indicate the mean value plus two standard deviations of stapes motion, *μ* + 2*σ*, before 0.16 ms. This shows that both BM and delayed stapes motion are above noise level at the same time.

Approximate distances from the stapes (see Methods) to the recording sites in Fig 2A and 2C equal 5.27 mm. Using that distance and a 4-μs travel time (signal-front delay), we computed a velocity estimate of 1317 m s^-1^. (A similar velocity estimate, 1450 m s^-1^, was obtained from the responses at location 2 in Fig 1.) These estimates are very close to the speed of sound in water and suggest that fast pressure waves do elicit BM motion.

Phase-vs.-frequency functions, *φ*(2*πf*), shown in Fig 2B and 2D (black continuous lines) were obtained from the BM responses displayed in Fig 2A and 2C, respectively, relative to the original stapes response. (Black dashed lines in Fig 2B and 2D represent phase functions expressed relative to umbo motion.) For *f* ≤ 1.5 * CF, there is a monotonic decrease, or phase lag, in *φ*(2*πf*). Phase plateaus are observed at higher frequencies, as indicated by the red dashed lines in Fig 2B and 2D. The corresponding slopes of each of the aforementioned lines are -6 and -1 μs, which are close in value to the slope indicated in Fig 1B. Delayed umbo responses (dashed lines in Fig 2A and 2C) appear to start before stapes and BM motion. Note, however, that BM phase responses expressed relative to the original umbo response (Figs 1A, 2B and 2C) also exhibit a high-frequency plateau.

The evidence for very short travel times shown in Figs 1 and 2 comes from the comparison between the initial segments of the vibration responses of the stapes and the BM. It is also instructive, however, to look at travel times between two adjacent locations along the BM in the same cochlea. The phase-vs.-frequency functions in Fig 2D were obtained in two locations in the same BM. The phase lags at CF are approximately the same as shown in that figure (open circles). Phase plateaus occur in both phase functions as shown in the main figure and in its inset, which contains the difference between the two curves [21].

**Fig 3:**
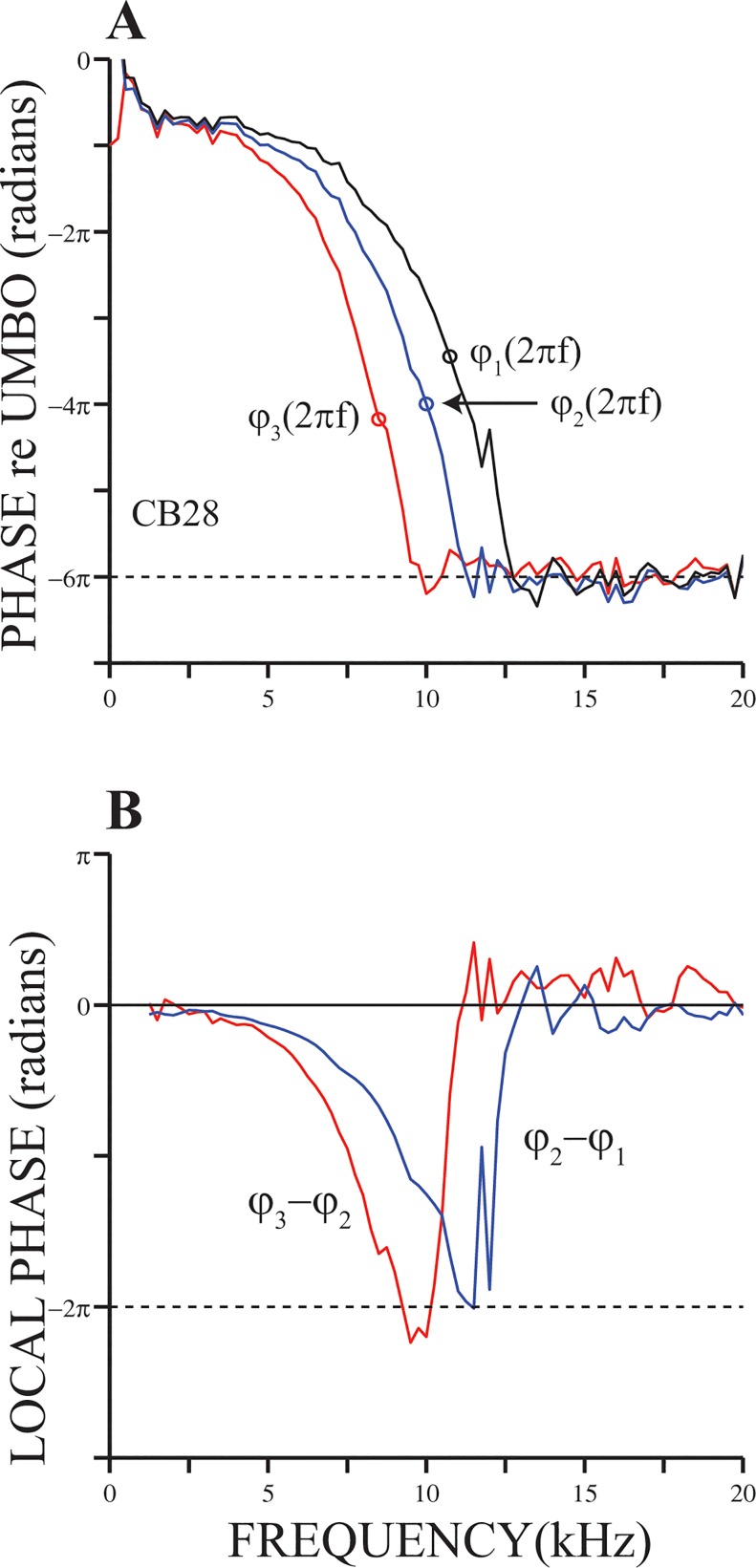
Local phase functions. Panel (A) displays plots of *φ*(2*πf*), obtained from BM responses to clicks at three locations along the same cochlea. Two local phase functions obtained from the phase functions in (A) are shown in panel (B).

Fig 3A shows *φ*(2*πf*) relative to umbo motion computed from click responses measured at three BM locations in the cochlea of another chinchilla. CFs of the locations (8.5, 10 and 10.75 KHz) are indicated next to each curve. The distance between sites 1 and 2, d_12_, equals 266.4 μm (see Methods section). Similarly, d_23_ = 598.7 μm. Phase curves in Fig 3A and those shown in Figs 1 and 2 have many similarities, including high-frequency phase plateaus. Phase lags in the plateau regions in Fig 3A are approximately three cycles relative to BM motion. That is, for frequencies in the plateau region, BM and malleus motions are approximately in phase (see also Figs 1C, 2B and 2D). Local phase functions [21], which are displayed in the inset in Figs 2D and 3B, also exhibit high-frequency plateaus. We see the existence of these plateaus as an indication that, in response to click stimuli, the BM starts to move in phase with neighboring locations.

### Time-domain gain functions

**Fig 4:**
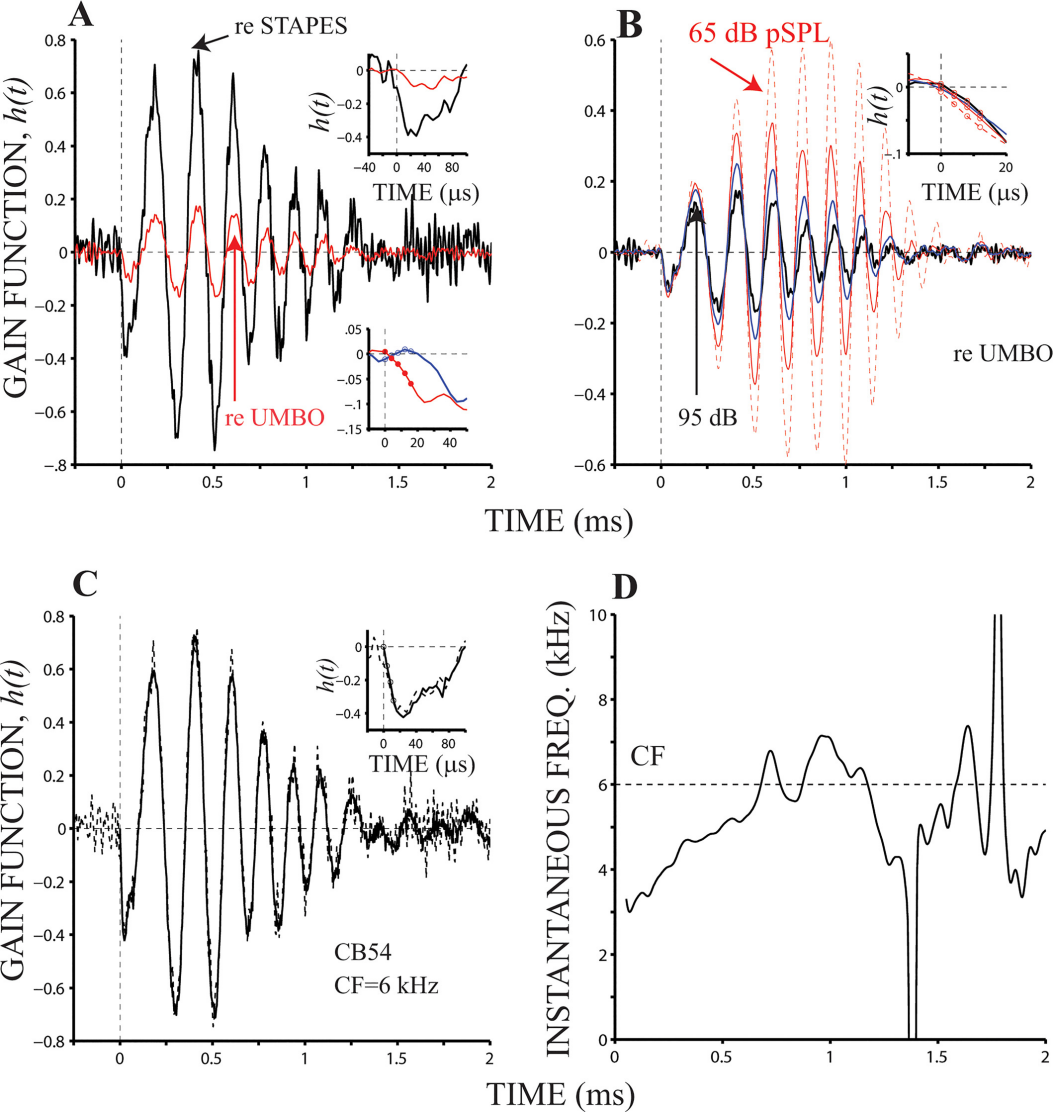
Instantaneous gain functions. Panel (A) displays *h*(*t*) functions obtained from middle ear (stapes or umbo) and BM responses to 95-dB pSPL clicks. The thickest lines in (B) were computed from responses to 95-dB pSPL clicks (the waveform is the same plotted in panel (A)); the remaining lines from responses to 85-, 75- and 65-dB pSPL clicks. All the traces in panel (B) were obtained using the umbo response as input. Upper insets in (A) and (B) show the initial part of the *h*(*t*) functions displayed in the main panels. Symbols in the inset in panel (B) represent the four initial samples of the gain functions. Panel (C) displays one *h*(*t*) function (black line) from panel (A) (95-dB pSPL) as well as a synthetized version (gray line) of it (see Eq 2 in Methods section). The blue line with symbols in the lower inset in panel (A) represents *h*(*t*) function evaluated after delaying the input waveform by 5 samples (see main text). The red line with symbols in the same inset is the same as the one shown in the upper inset (same color). Panel (D) exhibits the instantaneous frequency representation of the gain function in panel (A) (red line).

Fig 4A displays time-domain gain functions, *h*(*t*), obtained using stapes and umbo data (black and red lines, respectively) as the denominator in Eq 1. A three-point moving average filter was applied to *h*(*t*) functions in 4A. The first half-cycle of both functions is displayed in the upper inset in Fig 4A, where it is possible to observe a small signal-front delay (about 4 μs). In spite of the apparent pure delay between umbo and stapes motion, e.g., see insets in Fig 2A and 2C, *h*(*t*) functions in Fig 2A (black and red lines) exhibit approximately the same delay (4 μs).

Although the red traces in the upper and lower insets represent the same gain function, the blue and black lines in the insets do not. Blue lines in the lower inset in Fig 4A was obtained using a delayed version of the umbo data. (The delay equals 5 samples, or 20 μs.) This was done to verify the ability of our method to detect pure delays between the input and output data.

*h*(*t*) functions were also computed as a function of stimulus level in 10-dB decrements using the umbo response as input (Fig 4B). Because of the non-linear behavior of BM motion, amplitudes and shapes of all the waveforms in Fig 4B vary as a function of input level. (The thickness of the lines is in proportion to the intensity of the click.) Waveform amplitudes tend to increase as the stimulus level decreases, i.e., gain functions increase as the stimulus level decreases. These amplitude differences become more notable after the first half-cycle of *h*(*t*). The waveforms in Fig 4B computed using click levels ≤ 85 dB pSPL are shown after being processed with a zero-phase low-pass filter, with a cut-off frequency at 18 kHz. The *h*(*t*) function obtained using the 95-dB pSPL click in Fig 4B is the same as the one depicted with a red line in Fig 4A.

The inset in Fig 4B displays the onset of the *h*(*t*) functions also shown in the main panel. Each of the four dots in the inset represents *h*(*t*) values from t = 0 until t = 12 μs. These results show that BM motion begins within 4 μs after the onset of middle-ear vibrations. Even at a relatively weak stimulus level (65 dB pSPL), the value of the signal front delay does not change.

Estimates of show non-zero elements *h*(*t*) for *t* < 0 (Fig 4A and 4B), which were considered noise. (Negative times are represented in the second half of the vector returned by MATLAB’s *ifft* function.) To verify that the values shown for *h*(*t*) for *t* ≥ 0 do not anticipate middle-ear movement, a causality test was performed (see Methods).

Fig 4C displays one of the *h*(*t*) functions displayed in Fig 4A along with a *synthesized* version of *h*(*t*). (The waveform was computed from the responses to 95-dB pSPL clicks; the synthesized version of *h*(*t*) is depicted by a gray line in Fig 4C.) The synthesized version of *h*(*t*) was obtained by inverse Fourier transformation of a complex vector whose real part equals *X*(*ω*) in Eq 2 and imaginary part matches the imaginary part of *H*(*ω*). The inset in Fig 4C shows a version of *h*(*t*) from -20 to 100 μs. For *t* ≥ 0 the two waveforms are very similar and overlap almost completely, indicating the causality of *h*(*t*).

Plots of instantaneous frequency as a function of time of BM click response at the base of the cochlea (e.g., Fig 4 in [5] and Fig 5 in [11]) exhibit a representation of frequencies below CF near the response onset. Fig 4D displays a BM click response as well as its instantaneous frequency representation for the *h*(*t*) function depicted in Fig 3A using a red line. The figure shows that instantaneous frequency values during the first negative oscillation are, for example, below 5 kHz. BM responses to low-frequency stimuli appear before responses to stimuli above CF. It thus appear that the delays in BM responses to low-frequency stimuli are responsible for the very short latencies in click responses.

**Fig 5:**
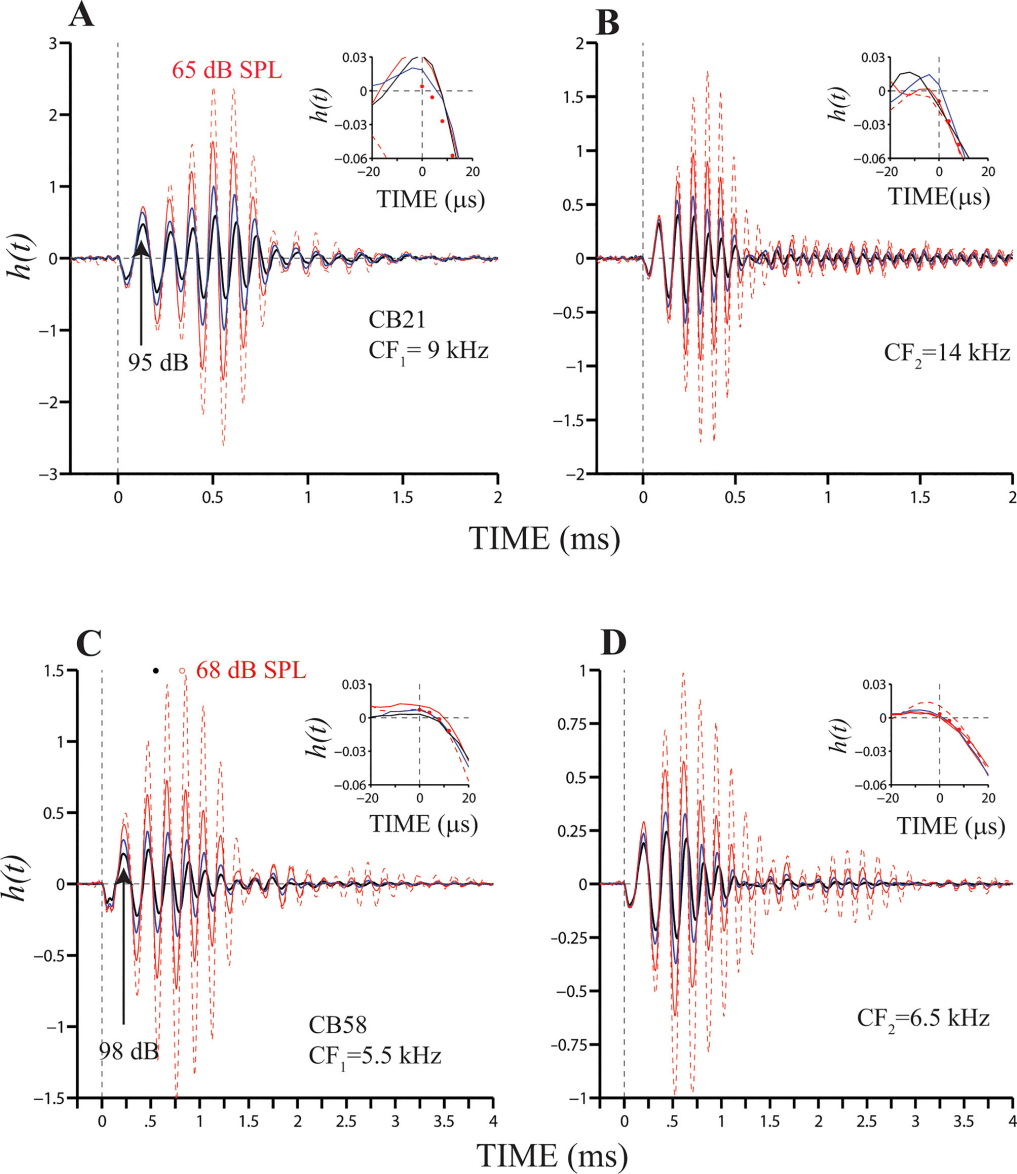
*h*(*t*) functions at several cochlear locations. Gain functions were computed from BM and umbo responses to clicks at four intensity levels. Results in (A) and (B) originate from data recorded at two location in the same cochlea. (Likewise for results in (C) and (D).) CFs and stimulus levels are shown in each panel. Insets display fragments of each gain function, from -20 to 20 μs. Red filled circles in each panel represent the average of each set of four *h*(*t*).

Fig 5 shows *h*(*t*) functions computed from BM responses in two animals. Responses were measured in each cochlea at two locations and expressed relative to umbo motion. Each of the waveforms were obtained at a given level, with the thickest continuous black line and the red dashed line indicating responses to the maximum and minimum stimulus levels, respectively. Insets in Fig 5 display the initial parts of *h*(*t*) functions shown in the main panels. Red dots in the insets represent the average value of all the functions. A common observation is that BM motion starts at approximately 4 μs, regardless the location at the base and the stimulus level. Because of the nonlinear effects with level of BM motion, the waveforms in Fig 5 do not overlap. In fact, overall amplitude values of the gain functions increase as the stimulus level decreases. Centers of gravities [22] of gain functions also shift towards later times as the stimulus level decreases, as shown in Fig 5C (filled and open circles). In that figure, *h*(*t*) functions have centers of gravities of 0.55 and 0.82 ms for click levels of 98 and 68 dB pSPL, respectively.

### Fast waves and the plateau region

The commonly named plateau is a frequency region above CF in which the amplitude or phase relation between middle ear and BM motions is frequency independent (e.g., red line in Fig 1C). Results of previous analysis in this work show that the 4-μs signal front delay, estimated from the analysis in Figs 1B, 2A and 2C, is similar to the group delay computed from phase data in the high-frequency plateau region. This suggests a relation between the fast vibration mode and BM responses in the plateau region, as demonstrated by Cooper and Rhode [23] in their experiments at the apex of the cochlea.

**Fig 6:**
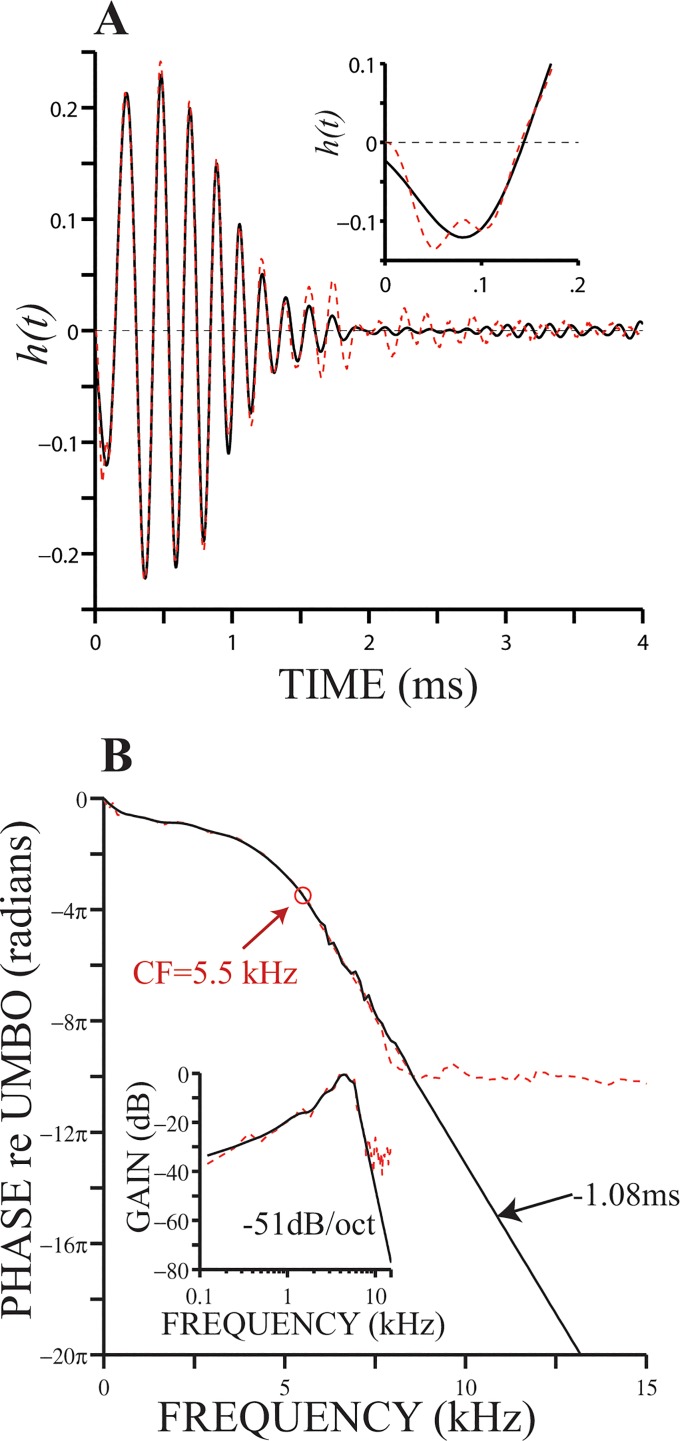
Gain function without frequency plateau. Panel (A) displays the original gain function (red lines) and the modified version (black lines) of the original waveform (see main text). The inset in panel (A) shows the onset of both gain functions. Modification of the original phase function was achieved by replacing the plateau with phase values having a group delay of 1.08 ms (black line in (B)). Original amplitude plateaus (red line in inset in (A)) were replaced with values that decrease at a slope of -51 dB per octave.

Fig 6A (red line) shows a gain function, which was previously displayed in Fig 4A and 4B, along with a plot of its onset (red line in the inset). Phase and amplitude functions—obtained using the Fourier transform function fft() in MATLAB—are respectively shown in the main panel in Fig 6B and its inset (red lines). Amplitude and phase plateaus are evident in the results in Fig 6B (red lines) for frequencies much higher than CF (i.e., > 9–10 kHz). The other waveform in Fig 6A, which is depicted with a black line, represents the impulse response of a filter whose amplitude and phase functions are shown using black lines in Fig 6B. We conclude that removing the amplitude and phase plateaus from the frequency representation and replacing them with new values, as depicted by the black lines in Fig 6B and its inset, has little effect on the resulting gain function (black lines in Fig 6A and the inset).

## Discussion

The onsets of BM responses to clicks measured at several locations along the base of the cochlea occur within 4 μs after the start of middle ear vibrations (Figs 1–5). Such signal-front delays are smaller than previously reported delays for the chinchilla [5, 11], which were around 20–32 μs. A concern with previously reported signal-front delays is that they were estimated using a method that depends on an arbitrary onset definition—a proportion of the maximum absolute value of the first oscillation of the response. Another criticism of that method is that it is difficult to separate, at least visually, the pure (signal-front) delay from the resonance build-up time of the cochlear filter.

The use of metrics, such as group or phase delays, from linear system theory must be justified when analyzing nonlinear responses, such as those originating from the cochlea. BM responses to 0-dBA clicks (that is, without attenuation), as in Fig 1, reveal mostly linear BM properties. In fact, responses to very loud clicks in live and postmortem preparations are similar [11]. Moreover, there are striking similarities among BM responses to tones, clicks and noise [5, 16], which one would not necessarily expect from a nonlinear system, due to the linearization effect of broadband stimuli.

Idealized cochlear responses to clicks typically contain substantial signal-front delays, which are usually larger than a few microseconds, followed by a filter’s impulse response (for example, see Fig 1A in [7]). Our results disagree with that conceptualization. Signal-front delays reported here correspond to those expected from a wave that propagates through the cochlea at the speed of sound in water and not from the “traditional” traveling wave as envisioned by von Békésy.

Theoretical discussions of waves generated at the oval window usually include two types of waves: the slow (pressure difference) and the fast (compression) waves [24, 25]. The pressure difference wave can be viewed as a traveling wave, which is responsible for BM motion. Fast compression waves, which propagate at the speed of sound in water, by definition cannot excite BM vibrations. Pressure differences in the most basal part of the cochlea have also been related to evanescent waves [26–28], which have fast modes as well and elicit BM motion. Published reports of BM motion allegedly associated with fast compression waves (e.g., [23,29]) are generally thought to be the result of experiment artifacts.

### Fast responses at the base of the cochlea

Plots of BM and stapes responses to clicks reveal that, after delaying the latter, the initial segments of both responses are very similar (Figs 1 and 2). This applies to all cases in which stapes and BM responses were compared. Similarly, signal-front delays of *h*(*t*) functions equal 4 μs at all recording locations along the first turn of the cochlea (Figs 4 and 5). At the cochlear location with CF = 5.5 kHz (Fig 5), the lowest CF recorded in our experiments, a 4-μs travel time is similar to the value expected for sound waves in water. We estimated a propagation velocity of 1505 m s^-1^ from the aforementioned signal-front delay and distances calculated using a cochlear map [14]. The newly computed velocity is much higher than an estimate (280 m s^-1^) of the speed of fast waves based on responses to clicks at the apex of the chinchilla cochlea. Those recordings were made through an opening of the otic capsule over scala vestibuli [23], whereas the present measurements were made via a hole overlying scala tympani. Whether this might explain the differences in estimates of the propagation velocities is unknown.

The fact that onset delays of time-domain gain functions, *h*(*t*), did not change as a function of CF (Fig 5) is intriguing because it implies that all basal regions of the cochlea start to move at approximately the same time, as hypothesized by Helmholtz [2]. Our findings, however, do not disprove the coexistence of a slow traveling wave.

### The plateau region

Rhode [30] first showed evidence of amplitude and phase plateaus in BM motion, which he argued were possibly a product of “another mode of vibration present in the cochlea.” Phase lags at frequencies well above CF tend to be relatively constant and in phase with malleus motion (Figs 1C, 2B, 2D, 3A and 6A), which agrees with Rhode’s original measurements of BM vibration for frequencies above CF (see also remarks in [25]). In the present work, group delay estimates in the plateau region and 4-μs signal-front delay estimates are similar.

BM vibration amplitudes expressed relative middle-ear motion in the plateau region are much smaller than similar ratios obtained at frequencies around CF. In Fig 6B, for example, gain values at the plateau region are more than 30 dB smaller than gains around 4–5 kHz. The difference in gain would certainly increase for low-level stimulus in a nonlinear preparation. Gains measured at frequencies below CF, e.g., around 2–3 kHz in Fig 6B, are approximately 10–20 dB below the maximum gain. It is, therefore, not surprising that only frequencies below CF are evident at the onset of BM click responses (Fig 4D), in spite of the very short group delays computed in the plateau region.

### Previous evidence of fast waves

Results of the analysis performed by Lighthill [25] on Rhode’s data suggested that BM motion at frequencies above CF, around the plateau region, are a consequence of fast compression waves. Models that include evanescent waves [26, 27] also exhibit phase plateaus.

Experimental evidence of fast waves in the time domain might have been shown in the BM responses to clicks recorded in Rhode’s original preparation [31], which sometimes exhibited “early” peaks (at ≈35 μs re malleus motion). Robles et al. [31] considered those results as indicators of “whole cochlea” movements evoked by high-intensity clicks. In the present work, however, we show that fast responses can be measured even at stimulus levels of intermediate intensity (Figs 4 and 5).

Additional evidence for fast waves at the apex of the chinchilla and guinea pig cochleae has also been reported [23, 29]. Cooper and colleagues were able to separate the fast and slow components of traveling waves (in the time domain) in their cochlear partition recordings. The amplitude of the fast traveling wave increased linearly with stimulus level and was substantially attenuated after sealing the optic capsule opening, leaving mostly intact the slow wave component of their results. Even with the tightest seals, however, the fast wave never disappeared [23, 29]. These results led to the suggestion [23] that “the fast response components may not exist in truly intact cochleae.” This statement is pertinent not only to the present work but to all published works of direct measurements of BM motion, which were performed in unsealed cochleae.

Fast compression (acoustic) waves have also been proposed by Ren and colleagues [32, 33]. Whereas our results indicate that BM motion begins with a delay consistent with the speed of sound in fluid, i.e., faster than the slow traveling wave, Ren’s results showed no involvement of the BM in the reverse propagation of oto-acoustic emissions. The findings by Ren’s group might be related, but this relationship would probably be complex.

### Are fast waves at the base the result of artifacts?

In their hydrodynamical theory of the cochlea, Peterson and Bogert [24] described two waves that travel along the cochlea: a fast common-mode wave, P+, which travels at the speed of sound in water, and a much slower differential wave, P-. By definition, only the P- wave has an effect on the BM. (The effect of P+ on BM motion is usually considered small or nonexistent [26, 34]). Perhaps because of this argument and the evidence in [23, 29], BM motions associated with fast waves are usually thought to be artifacts.

It is possible that BM responses measured at the base of the cochlea consist of fast and slow components, just as in the apex [23, 29]. Separating the fast and slow components in our recordings, however, might be more challenging than in the apex because of the faster travel times of the two components. It thus remains to be proven whether the short signal-front delays measured for this work are due to an experimental artifact. Two lines of evidence tend to negate this possibility. First, latencies of responses to rarefaction clicks of ANFs innervating intact (and thus sealed) cochleae are very short and exhibit little variation among units with CFs >8–9 kHz (see Fig 10A in [3]). Those recordings are consistent with the notion that BM along the entire cochlear base moves synchronously. (Evidence of fast waves in ANFs in the form of response plateaus in their tuning curves has also been shown recently [35].) Second, the proximity to the round window of the recording sites at the first turn of the cochlea, “where any effects on the cochlea’s hydrodynamics should be minimized by the presence [of the window]” (see [29] and references therein), probably implies that the effects of not sealing the cochlea on the measurements presented here are minimal. There is also the possibility of the existence of evanescent waves, which as previously indicated, have a fast mode and elicit BM motion. We also argue that, at least in the chinchilla, BM recordings at locations with CFs ≥ 11–12 kHz are usually performed through the round window—without the need of a cochleostomy. All this evidence suggests that the fast mode of BM vibrations occurs in intact preparations.
